# Process development for pandemic influenza VLP vaccine production using a baculovirus expression system

**DOI:** 10.1186/s13036-019-0206-z

**Published:** 2019-10-23

**Authors:** Chia-Chun Lai, Yu-Chieh Cheng, Pin-Wen Chen, Ting-Hui Lin, Tsai-Teng Tzeng, Chia-Chun Lu, Min-Shi Lee, Alan Yung-Chih Hu

**Affiliations:** 1 National Institute of Infectious Diseases and Vaccinology, NHRI, 35 Keyan Road, Zhunan, Miaoli County, 35053 Taiwan; 2 College of Life Science, National Tsing Hua University, 101, Section 2, Kuang-Fu Road, Hsinchu, 30013 Taiwan

**Keywords:** Influenza vaccines, Virus-like particle, Dissolved *oxygen*

## Abstract

**Background:**

Influenza viruses cause hundreds of thousands of respiratory diseases worldwide each year, and vaccination is considered the most effective approach for preventing influenza annual epidemics or pandemics. Since 1950, chicken embryonated eggs have been used as the main method for producing seasonal influenza vaccines. However, this platform has the main drawback of a lack of scale-up flexibility, and thus, egg-based vaccine manufacturers cannot supply sufficient doses within a short period for use for pandemic prevention. As a result, strategies for reducing the manufacturing time and increasing production capacity are urgently needed. Non-virion vaccine methods have been considered an alternative strategy against an influenza pandemic, and the purpose of maintaining an immunogenic capsule structure with infectious properties appears to be met by the virus-like particle (VLP) platform.

**Results:**

An influenza H7N9-TW VLP production platform using insect cells, which included the expression of hemagglutinin (HA), NA, and M1 proteins, was established. To scale up H7N9-TW VLP production, several culture conditions were optimized to obtain a higher production yield. A high level of dissolved *oxygen (DO)* could be critical to H7N9-TW VLP production. If the DO was maintained at a high level, the HA titer obtained in the spinner flask system with ventilation was similar to that obtained in a shake flask. In this study, the HA titer in a 5-L bioreactor with a well-controlled DO level was substantially improved by 128-fold (from 4 HA units (HAU)/50 μL to 512 HAU/50 μL).

**Conclusions:**

In this study, a multigene expression platform and an effective upstream process were developed. Notably, a high H7N9-TW VLP yield was achieved using a two-step production strategy while a high DO level was maintained. The upstream process, which resulted in high VLP titers, could be further used for large-scale influenza VLP vaccine production.

## Background

Influenza virus infections often cause human respiratory symptoms and result in public health concerns regarding seasonal and endemic infections and even unpredictable pandemic outbreaks. Currently, the most effective method for preventing influenza infection caused by the influenza virus is vaccination. Most influenza vaccines are manufactured using split antigen, inactivated whole-virion, or live attenuated method [1]. Although these vaccines exhibit high efficacy, biosafety concerns regarding the use of highly pathogenic avian influenza (HPAI) during the manufacturing process are an issue, and thus, the development of safer vaccines is needed. Virus-like particles (VLPs) are produced through the self-assembly of essential viral structural proteins expressed in a cell and exhibit morphological and structural features that are similar to those of native viruses. Because VLPs do not contain any infectious genetic materials, these particles are safer than whole-viral vaccines [2] and are thus considered a safe vaccine platform [3]. Recent evidence suggests that VLPs constitute a vaccine platform technology with high potential for use for a wide range of infectious viruses [4–6]. VLP vaccines have been produced using different expression systems, including bacterial, yeast, insect, mammalian, and plant systems [7–10]. Strategies to decrease the response time and increase the productivity of vaccines are urgently needed for pandemic preparedness. One of the most promising approaches is the production of VLP-based vaccines using the baculovirus expression vector system (BEVS) [11], which provides a rapid and efficient method to generate multiple recombinant proteins for the formation of VLPs. To date, several established insect cell lines have been shown to be susceptible to baculovirus infection [11–13]. Previous studies [14–16] have shown that a number of factors can influence protein expression, and these include the production media, the level of dissolved *oxygen (DO)*, the virus multiplicity of infection (MOI) and the time point after infection used for harvesting. Notably, these established insect cell lines show variable abilities to amplify baculovirus and express soluble protein. In addition, these previous studies have suggested that different production conditions support the production of varying levels of protein expression [17, 18]. Therefore, optimal culturing conditions are essential for VLP production.

Over the past 10 years, HPV vaccines based on VLP production techniques using insect cells (Cervarix®, GSK) have been licensed by the U.S. FDA [19–21]. This vaccine can protect against cervical and anogenital infections and diseases. The development of a vaccine production system using insect cells has attracted the attention of many scientists [5, 6, 10, 22–24]. The influenza VLP structure could be self-assembled by different hemagglutinin (HA), NA, and M1 proteins, which have been shown to antagonize the threat of new influenza pandemics and increase the flexibility of their manufacturing [7, 22, 25, 26]. The BEVS was used to develop an influenza VLP vaccine by Novavax, Inc. [27–29] and a recombination influenza subunit protein by the *Protein Sciences Corporation* [30, 31] to improve the response time for influenza pandemic preparedness, and these studies have demonstrated that insect cell culture-based manufacturing has been accepted in the influenza vaccine industry.

A recent H7N9 influenza virus outbreak occurred in China, and recent cases have also been reported in Taiwan [32, 33]. Hence, in Taiwan, the H7N9 influenza virus poses health risks and might lead to an outbreak. In this study, we developed a H7N9-TW VLP production method using BEVS and used this method to generate a multigene expression vector for coexpressing the essential components (H7, N9, and M1 from the Influenza A/Taiwan/1/2013 (H7N9) strain) of VLPs. The production yield of H7N9-TW VLPs using two different insect cell lines, Sf-21 cells and High Five™ cells, was compared, and the advantages of the newly developed process development strategy were combined with those of these two insect cell lines. First, we prepared the virus stock using Sf-21 cells based on their notably high virus-propagation ability and then infected High Five™ cells for H7N9-TW VLP production. The culture conditions and the upstream process of VLP production were then optimized, and the scalability from a 500-mL spinner flask to a 5-L bioreactor was also studied.

## Results and discussion

### Establishment of the H7N9-TW VLP expression system

**Fig. 1:**
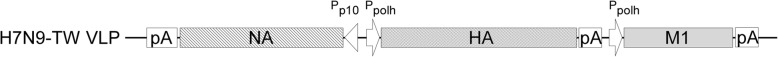
Production of influenza H7N9-TW VLPs in the baculovirus expression vector system. cDNAs of the HA, NA, and M1 proteins were derived from the sequences of the Influenza A/Taiwan/1/2013 (H7N9) virus strain by polymerase chain reaction (PCR). The amplified DNA fragments were subcloned into the corresponding sites in a baculovirus expression vector

**Fig. 2:**
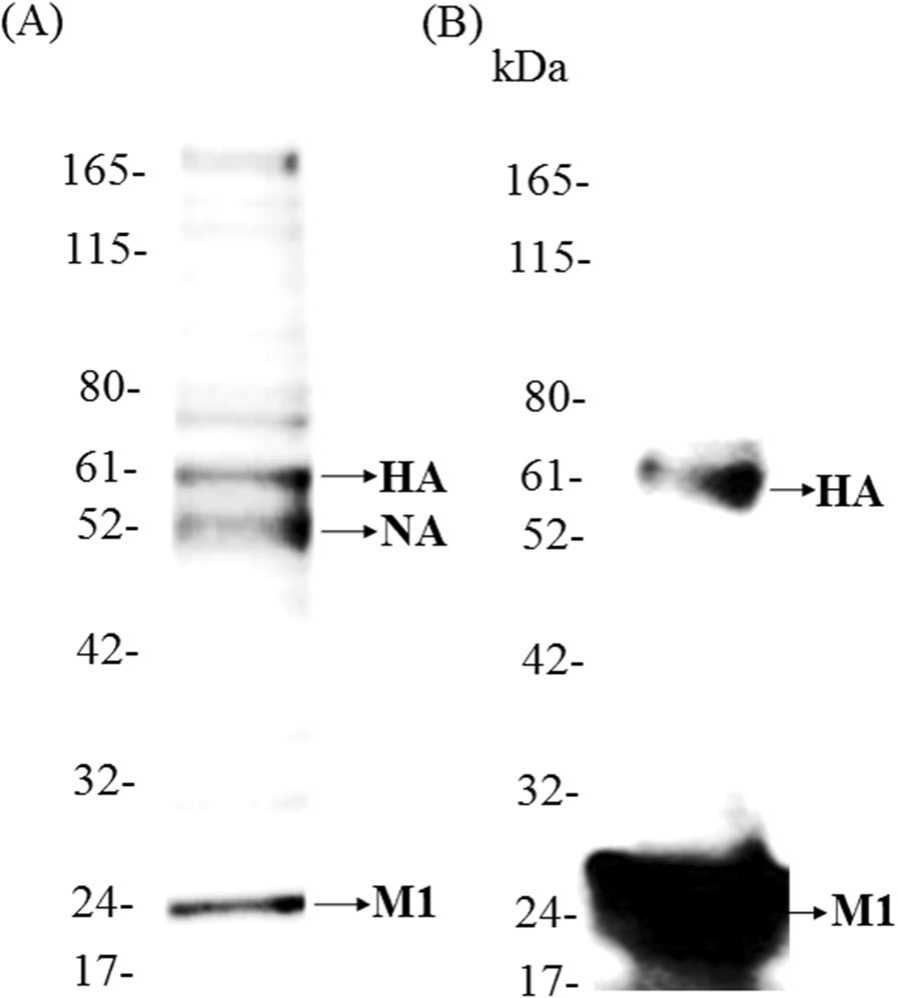
Diagram of the development of the upstream process. The upstream process combined a two-step strategy. The baculovirus was first amplified in Sf-21 cells to establish the virus stock, the baculovirus stock was subsequently used to infect High Five™ cells for the production of H7N9-TW VLPs

The HA, NA, and M1 genes from the Influenza A/Taiwan/1/2013 (H7N9) strain were cloned into the pFastBac DUAL vector (Invitrogen, USA) (Fig. 1). The resultant plasmid (H7N9-TW VLP) was employed to generate the recombination baculovirus for influenza VLP expression using the Bac-to-Bac® system (Invitrogen) [11]. The recombination baculovirus was successfully established in a BEVS. To identify suitable cell lines for H7N9-TW VLP production, the cell growth ability of Sf-21 and High Five™ cells was compared. The Sf-21 cells were cultured in Sf-900™ II SFM with an initial seeding cell density of 2 × 10^5^ cells/mL, and their cell density reached 1.48 × 10^6^ cells/mL after 3 days (corresponding to a cell doubling time of 33.32 h). In addition, the High Five™ cells were cultured in Express Five® SFM with an initial seeding cell density of 2 × 10^5^ cells/mL, and their cell density reached 3.30 × 10^6^ cells/mL after 3 days (corresponding to a cell doubling time of 18.30 h) (Table 1). These data showed that High Five™ cells exhibit improved growth than Sf-21 cells. In addition, the baculovirus titer generated with the Sf-21 cells (1 × 10^8^ virions/mL) was higher than that generated with the High Five™ cells (3 × 10^6^ virions/mL) (Table 1). These data showed that the Sf-21 cells exhibited higher baculovirus-production ability than the High Five™ cells. The HA titers of H7N9-TW VLPs generated using these two cell lines were compared. The HA titer of the H7N9-TW VLPs produced using Sf-21 cells did not exceed 64 (HA units (HAU)/50 μL), whereas the High Five™ cells produced H7N9-TW VLPs with an HA titer of 512 (HAU/50 μL) (Table 2), which demonstrated that H7N9-TW VLPs are more easily produced by High Five™ rather than Sf-21 cells. Previous studies revealed that the transfection of different insect cell lines with the same plasmid might result in various levels of protein expression and glycosylation [9, 34]. Therefore, High Five™ cells were selected as a host cell candidate for the production of influenza H7N9-TW VLPs using the BEVS system. Based on the above-described data, the best strategy for the development of this process comprised baculovirus replication in the Sf-21 cells and H7N9-TW VLP production in the High Five™ cells. This two-step strategy was not only beneficial for baculovirus and H7N9-TW VLP production but also decreased baculovirus contamination, which simplified downstream purification (Fig. 2 and Additional file 1: Table S1).

**Table 1:** Characteristics of Sf-21 and High Five™ cell growth and baculovirus virus and H7N9 TW-VLP production in a 300-mL shake flask

Cell line	Sf-21 cell	High Five™ cell
Medium	Sf-900™ II SFM	Express Five® SFM
Seeding cell density (cells/mL)	2.0 × 10^5^	2.0 × 10^5^
Cell doubling time (hour)^a^	33.3	18.3
Harvested cell density (cells/mL)	1.5 × 10^6^	3.3 × 10^6^
Fold increase of cell density	7.4	15.3
Baculovirus virus (virion/mL)^b^	1.0 × 10^8^	3.1 × 10^6^
HA titer (HAU/50 μL)	64	512

^a^The cell doubling time was calculated using the following formula: DT = time (T) x ln2/ln (Xe/Xb), where T is the incubation time in any units, Xb is the cell number at the beginning of the incubation period, and Xe is the cell number at the end of the incubation period

^b^The baculovirus titer (virion/mL) was calculated using the TCID_50_ method

**Table 2:** Comparison of High Five™ cells cultured in 300-mL shake flasks with different commercially available culture media

Name of commercial medium	Seeding cell density (cells/mL)	Harvested cell density (cells/mL)	Cell doubling time (hour)	Fold increase of cell density	HA titer (HAU/50 μL)
HyClone™ SFM4Insect	2.00 × 10^5^	2.60 × 10^6^	19.46	13.00	512
EX-CELL® 405	2.00 × 10^5^	1.80 × 10^6^	22.71	9.00	256
Esf-921	2.05 × 10^5^	2.25 × 10^6^	20.83	10.98	256
Express Five® SFM	2.16 × 10^5^	3.30 × 10^6^	18.30	15.28	256

### Protein composition and functional analysis of influenza H7N9-TW VLP

**Fig. 3:**
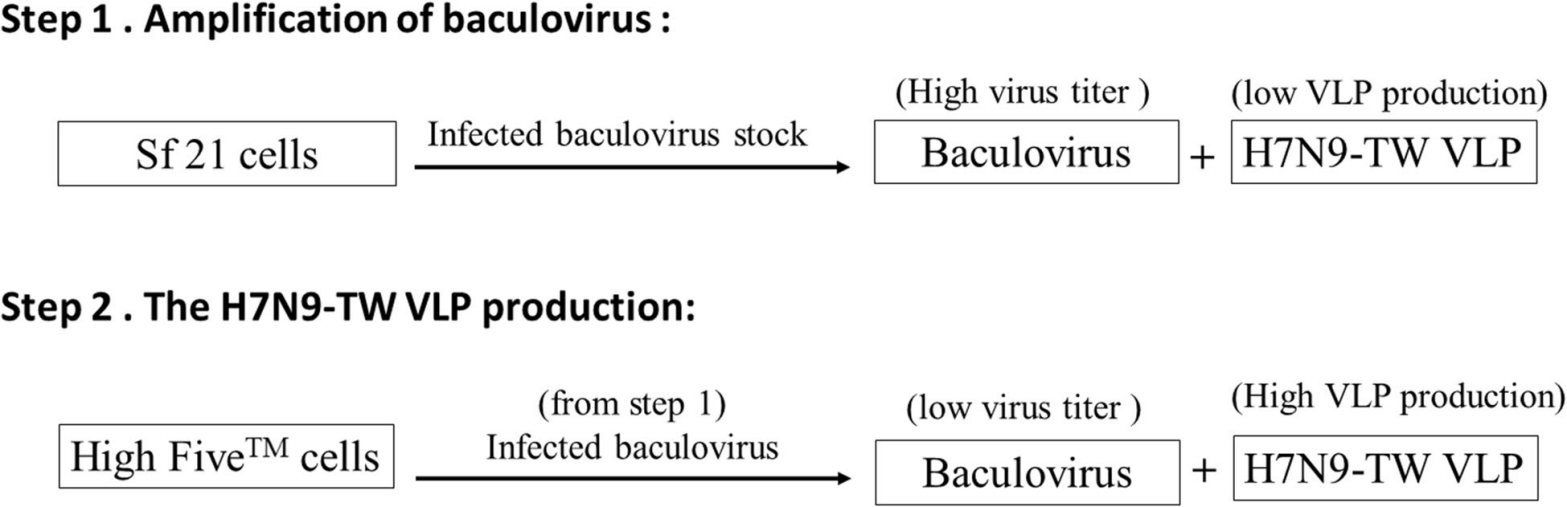
Analysis of H7N9-TW VLP expression by **a** SDS-PAGE and **b** western blotting. High Five™ cells were infected with the baculovirus, and at 3 days postinfection, the resulting H7N9-TW VLPs were harvested and analyzed

The protein composition of H7N9-TW VLPs derived from High Five™ cells was analyzed by SDS-PAGE and western blotting. The levels of HA (58 kDa), NA (48 kDa), and M1 (24 kDa) proteins were measured (Fig. 3a), and the composition of H7N9-TW VLPs was similar to that obtained in a previous study [35]. In addition, the HA and M1 proteins in H7N9-TW VLPs were recognized by the ferret anti-H7N9 antiserum generated in our previous study [36] (Fig. 3b). The neuraminidase activity of the NA protein was analyzed, and the results showed that the enzymatic activity of the NA protein was approximately 17 U (nmol/hour/mL). These protein analyses indicated that the H7N9-TW VLP plasmid can be used to successfully express HA, NA and M1 proteins in a BEVS.

### Establishment of influenza H7N9-TW VLP production conditions

**Fig. 4:**
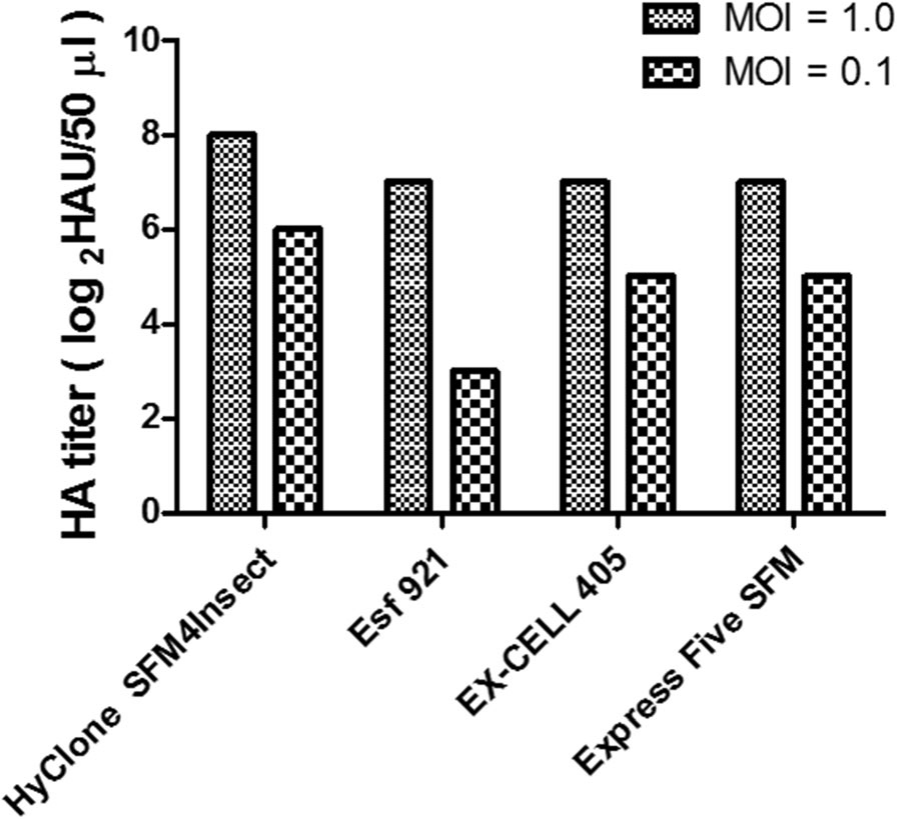
Comparison of HA titers obtained with two different MOIs in various commercial media. High Five™ cells were separately infected with the baculovirus at an MOI of 0.1 and 1 in a 300-mL shake flask and harvested at 3 days postinfection

With respect to upstream process development, serum-free media are essential for VLP production. The productivity levels of H7N9-TW VLPs derived from High Five™ cells cultured with different media (HyClone™ SFM4Insect, EX-CELL® 405, Esf-921, and Express Five® SFM) in 300-mL shake flasks were analyzed, and the results showed that the best HA titers in H7N9-TW VLPs generated with High Five™ cells were obtained by culturing in HyClone™ SFM4Insect medium (Table 2). Specifically, the average HA titer was 256 HAU/50 μL. These data also showed that the nutrient composition of the medium affects the H7N9 influenza VLP levels, and the data were in good agreement with those obtained in previous studies [17]. Thus, in the subsequent experiments, the H7N9-TW VLPs were generated using High Five™ cells in HyClone medium. According to a previous study [14], the MOI is a critical factor in VLP production. Hence, the effect of the MOI on the production of the H7N9-TW VLPs using High Five™ cells cultured in HyClone™ SFM4Insect medium was monitored. In this study, High Five™ cells at a cell density of 2.00 × 10^6^ cells/mL were infected with baculovirus at an MOI of 0.1 and 1.0. The HA titer of the VLPs generated from an MOI of 0.1 did not exceed 32 (HAU/50 μL) in all tested media, and the HA titer obtained with an MOI of 1 reached approximately 256 (HAU/50 μL). Thus, the data suggested that a high MOI could enhance the production of H7N9-TW VLPs in the BEVS (Fig. 4).

### Optimization of H7N9-TW VLP production conditions

**Fig. 5:**
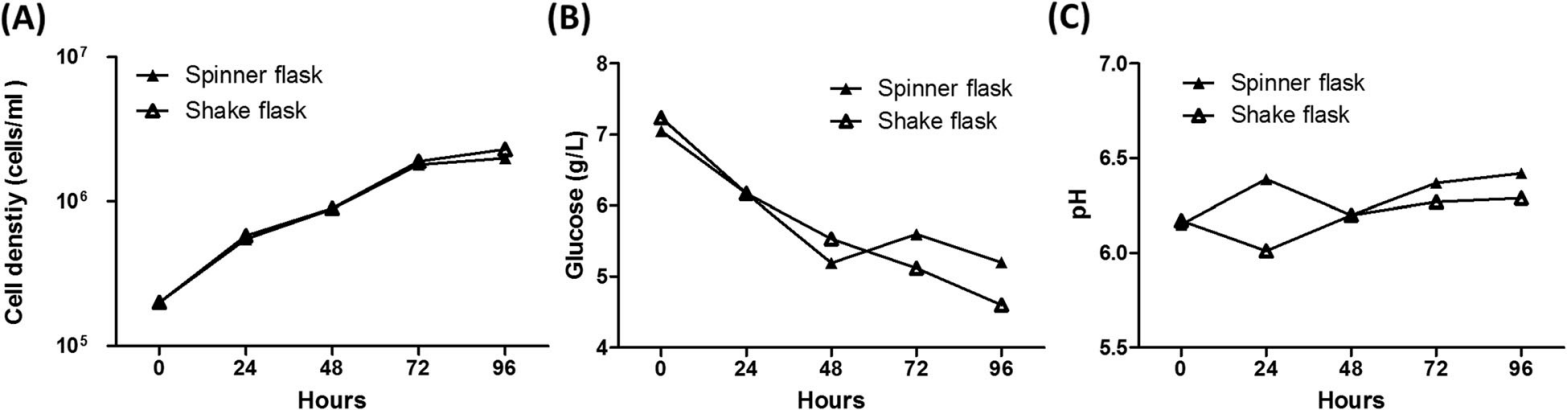
Cell growth profile and metabolic analysis of influenza H7N9-TW VLPs obtained using High Five™ cells in spinner flasks and shake flasks. High Five™ cells were cultured in 500-mL spinner flasks and 300-mL shake flasks. The following culture conditions were monitored throughout the experiment: cell growth profile (**a**), glucose utilization profile (**b**), and the pH profile (**c**)

**Fig. 6:**
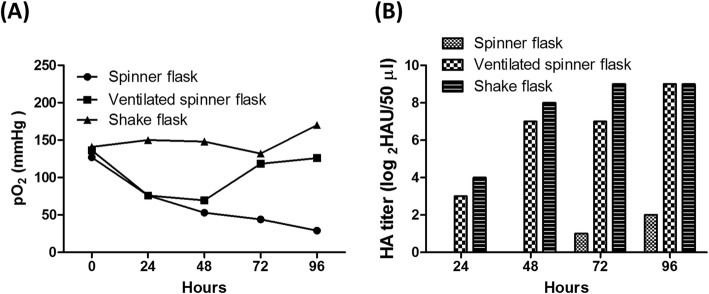
Influence of dissolved oxygen on H7N9-TW VLP production. High Five™ cells were cultured in 500-mL spinner flasks, spinner flasks, and shake flasks with air ventilation (100 mL/min) and infected with baculovirus. The DO concentration profile (**a**) and H7N9-TW VLP production profile (**b**) were monitored during the upstream process

For scale-up, efficiency and controllable production are crucial issues. The shaker flask is limited by a low working volume-to-flask volume ratio. Specifically, the use of a shaker flask requires more space for culture, and the transfer of culture conditions from shake flasks to benchtop bioreactors is challenging. However, the spinner flask system is usually considered for use in pilot studies aiming to scale-up production to a benchtop bioreactor because the culture conditions used in these two systems are highly similar [37, 38]. Therefore, the culture systems in shake and spinner flasks for H7N9- TW VLP production were compared. The total cell number of both systems increased from 2 × 10^5^ cells/mL to 2 × 10^6^ cells/mL during 3 days of culture. The metabolic parameters, including the total cell number, glucose consumption, and pH were similar between these two systems (Fig. 5). Surprisingly, a considerable difference in the DO level was observed (Fig. 6a). The DO level in the 500-mL spinner flask during the production period decreased from 150 to 30 mmHg, whereas a DO level of approximately 100 to 150 mmHg was detected in the 300-mL shaker flasks during the production period. This could result from the lower gas-liquid oxygen transfer in the gentle-mixing spinner-flask whereas the shake flask has higher gas-liquid oxygen transfer by higher agitation speed. To confirm this phenomenon, the experiment was performed in 500-mL spinner flasks with a small air pump that continuously filtered-air supply to the headspace (ventilated spinner flask). The DO levels in this system were similar to those in the shake flask system (Fig. 6a). Additionally, analysis of the H7N9-TW VLP yield showed that an HA titer of 512 HAU/50 μL was obtained with this system, and a similar titer was obtained with the 300-mL shake flask (Fig. 6b). In contrast, analysis of the yield of H7N9-TW VLPs obtained with the 500-mL spinner flask showed that the HA titer ranged from only 2 to 4 HAU/50 μL. The results showed that the DO level played a critical role in the production of H7N9-TW VLPs. Previous studies revealed that the DO level in insect cells could affect both the protein expression levels [39] and the production of foreign protein by insect cells [40]. These studies provide possible reasons explaining why H7N9-TW VLP production was affected by the DO level in this study.

### Evaluation of the scale-up feasibility of the process

**Fig. 7:**
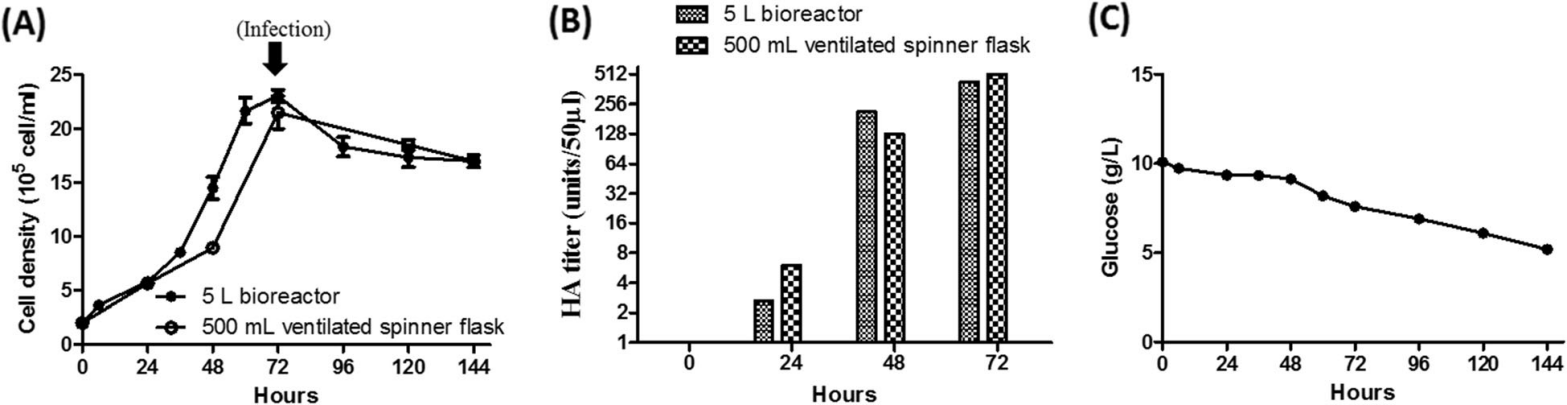
Comparison of the scalability of the upstream process from a 500-mL ventilated spinner flask to the 5-L bioreactor system. High Five™ cells were cultured in a 500-mL ventilated spinner flask and a 5-L bioreactor system, and the DO concentration was maintained at 80% throughout the production period. Once the Sf-21 cell density reached 2 × 10^6^ cells/mL, the cells were infected with the baculovirus. The cell growth curve (**a**), the H7N9-TW VLP production profile (**b**), and the glucose utilization profile (**c**) were monitored during the upstream process

According to the culture parameters found for High Five™ cells in 500-mL ventilated spinner flasks, the High Five™ cells were inoculated at a cell density of 2 × 10^5^ cells/mL and cultured in a 5-L bioreactor system with HyClone™ SFM4Insect medium. The cell density in this bioreactor system reached 2.42 × 10^6^ cells/mL after 3 days, and the cell growth profile was similar to that obtained with the 500-mL ventilated spinner flask (Fig. 7a). High Five™ cells were infected with baculovirus at an MOI of 1 in the 5-L bioreactor, and the viability of the High Five™ cells declined by 20 to 30% during the infection (Fig. 7a). The culture conditions for High Five™ cells in this 5-L bioreactor system, including the pH, glucose consumption, and DO, were also evaluated. During the culture period, the pH was maintained at 6.4; the initial glucose concentration decreased from an initial concentration of approximately 10.0 g/L to approximately 50% (from 10.0 g/L to 5.0 g/L) (Fig. 7c); and the DO was maintained at approximately 80% (equivalent to 150 mmHg). For H7N9-TW VLP production, High Five™ cells were harvested on day 3, and the HA titer of VLPs was 512 HAU/50 μL (Fig. 7b), which was similar to that obtained from VLPs produced at the pilot scale (500 mL). These results demonstrated that H7N9-TW VLP production can be scaled up to a 5-L bioreactor system and that this process exhibits good linear scalability Additional file 2: Table S2.

Furthermore, the level of DO, which is the critical parameter for H7N9-TW VLP production, in the 5-L bioreactor was maintained at 30% (equivalent to 50 mmHg) or 80% (equivalent to 150 mmHg) (Table 3, groups 1 and 3), and the growth profiles of the cells did not show differences between these two conditions. However, the HA titer was substantially improved by 128-fold in the 80% DO group compared with the 30% DO group. In addition, we also used a high cell density (approximately 8 × 10^6^ cells/mL) and a low level of DO (30%) for H7N9-TW VLP production in the 5-L bioreactor and obtained an HA titer of H7N9-TW VLPs that was less than 2 HAU/50 μL (Table 3, group 2). These data meet our expectations, which indicated that the DO level is indeed a critical factor affecting the VLP production yield in the upstream process and showed that the maintenance of a DO level of approximately 150 mmHg could enhance H7N9-TW VLP production. This VLP vaccine production platform not only determines the critical factors for enhancing the product yield but also reduces the biosafety concerns related to the manufacturing of vaccines [3, 41].

**Table 3:** Comparison of H7N9-TW VLP production in a 5-L bioreactor with different control parameters

Parameter/Group	1	2	3
Cell density (10^6^ cells/mL)^a^	2.0	8.0	2.4
DO (%)	30	30	80
pH	6.4	6.4	6.4
Agitation rate (rpm)^b^	120–180	120–180	120–180
HA titer (HAU/50 μL)	8	< 2	512

High Five™ cells were cultured in a 5-L bioreactor with different control parameters, infected with the baculovirus and harvested at 3 days postinfection

^a^The cell density value indicates the cell density at which baculovirus infection was performed

^b^The agitation rate was adjusted depending on the cell density during culture

## Conclusions

An H7N9-TW VLP expression platform using a BEVS was established, and the culture conditions were improved to obtain higher HA titers. This strategy involves the use of two insect cell lines, Sf-21 and High Five™ cells, and exhibits excellent performance for H7N9-TW VLP production, and the results of this study also demonstrate the optimal conditions for more rapid and efficient influenza VLP vaccine manufacturing. This study not only revealed improvements to not only the upstream process conditions but also found the critical parameter for enhancing the H7N9-TW VLP production yield. The improved upstream process for VLP vaccine production, which involves a two-step strategy, a high MOI, and a well-controlled level of dissolved *oxygen*, was successfully scaled up to a bioreactor system, and thus, the newly developed process could be easily scaled up to an industrial level in the future.

## Methods

### Cell culture and media

Sf-21 cells (Invitrogen, USA) were cultured in Sf-900II serum-free medium (Gibco, USA) for baculovirus production. For H7N9-TW VLP production, High Five™ cells (Invitrogen, USA) were cultured in various serum-free, non-animal-derived nutrient media, including Sf-900™III-SFM (Gibco, USA), HyClone™ SFM4Insect medium (GE Healthcare, USA), Insect-XPRESS™ medium (Lonza, Switzerland), and Express Five® SFM (Gibco, USA) supplemented with 9 mM L-glutamine. These insect cell lines were cultured at 27 °C.

### Preparation of recombinant baculoviruses

The pFastBac-p10-2polh promoter included one p10 promoter and two polh promoters bidirectionally, and each promoter had a polyadenylation [poly(A)] signal. The gene fragment of the second polh promoter and sv40 poly(A) were amplified from the pFastBac DUAL vector (Invitrogen, USA) by polymerase chain reaction (PCR) and cloned into the NotI/AvrII sites of the pFastBac DUAL vector to generate pFastBac-p10-2polh. The NA, HA, and M1 gene fragments were obtained by PCR from cDNA isolated from the Influenza A/Taiwan/1/2013 (H7N9) strain and cloned into the KpnI site under the control of the p10 promoter, the RsrII site under the control of the first polh promoter, and the XbaI site under the control of the second polh promoter in pFastBac-p10-2polh, respectively, to generate pH7N9-TW VLPs. The resulting plasmid was employed to generate H7N9-TW VLPs using the Bac-to-Bac® system (Invitrogen, USA) [11]. The baculoviruses were propagated by infecting Sf-21 cells (Invitrogen, USA) cultured in Grace’s insect basal medium (Invitrogen, USA) supplemented with 10% FBS (Gibco, USA) and harvested for H7N9-TW VLP production.

### Shake and spinner flask cultures of high five™ cells

The cultures were performed in 1-L shake flasks with a working volume of 300 mL. Each flask was inoculated with the cells at a density of approximately 2 × 10^5^ cells/mL. Once the High Five™ cell density reached 2 × 10^6^ cells/mL, the cells were separately infected at MOI values of 0.1 and 1. The H7N9-TW VLPs were harvested 3 days postinfection (dpi) by low-speed centrifugation at 4000 x g and 4 °C for 30 min, and the H7N9-TW VLP yield was then determined through an HA assay. Additionally, the cultures were performed in 1-L spinner flasks with a working volume of 500 mL and the same initial cell density and infection time that were used for the shake flask cultures. The cells were infected with the baculovirus at an MOI of 1, and the culture temperature during the upstream process were maintained at 28 °C. In addition, the level of DO was monitored during the process, and the H7N9-TW VLP production level was determined by HA titration.

### SDS-PAGE and western blot analysis

Approximately 1 μg of total protein from purified H7N9-TW VLPs was separated by 10% SDS-PAGE gels and stained using a Colloidal Blue staining kit (Invitrogen, USA). A western blot analysis was also performed as described previously [42]. The SDS-PAGE gel was electroblotted to a PVDF membrane, and this membrane was then blocked overnight with 5% nonfat milk and incubated with ferret anti-H7N9 antibody [36] (1:1000) in PBS with 0.1% Tween 20 (PBST) for 1 h at room temperature (RT). The membrane was then incubated with goat anti-ferret IgG (HRP) (1:10,000, Abcam, USA) in PBST for 1 h at RT. The blots were developed using the Luminata™ Crescendo Western HRP substrate (Millipore, USA), and images were captured using the Amersham Imager 600 system (GE Healthcare, USA).

### Assay of NA activity

The NA activity assay as performed using the NA-Fluor™ Influenza Neuraminidase assay protocol (Applied Biosystems, USA). The H7N9-TW VLPs were mixed with 2-(4-methylumbelliferyl)-a-D-N-acetylneuraminic acid to a final concentration of 100 μM and incubated at 37 °C on a shaker for 40 min, and the reaction was stopped by the addition of stop solution. Fluorometric measurements were performed immediately using a SpectraMAX M2 Reader with an excitation wavelength of 365 nm and an emission wavelength of 450 nm [43].

### Virological assays

HA titration was conducted in 96-well microplates using 0.5% turkey red blood cells following standard protocols [44]. The virus infectious titers were measured by assaying the 50% tissue culture infectious dose (TCID_50_) based on the cytopathic effect in Sf-21 cells [45].

### Production of H7N9-TW VLPs in the bioreactor

A 5-L bioreactor (NBS, USA) was used based on the same principles as the 500-mL ventilated spinner flask. The culture conditions were set using a BioFlo 310 controller (NBS, USA). Throughout the culture period, the culture was mixed with a three-pitch blade impeller at 120 to 180 rpm, the pH value was maintained at 6.4, and the temperature was maintained at 28 °C. Once the desired cell concentration was reached, High Five™ cells were infected with the recombination baculovirus at an MOI of 1, and the cells were harvested when the hematogluttin HA titer peaked. During the culture period, the HA titer, which was considered to reflect the level of H7N9-TW VLP production, was monitored, and the concentration of glucose in the culture supernatant was measured offline using a NOVA BioProfile 400 biochemical analyzer (Nova Biomedical Corporation, USA).

## Supplementary information

**Additional file 1: Table S1.** Comparison of influenza VLP production by different insect cells.

**Additional file 2: Table S2.** Comparison of influenza VLPs production with shake flask and Bioreactor.

## Data Availability

Not applicable.

## Abbreviations

BEVS: Baculovirus expression vector system
*DO*: Dissolved *oxygen*
*HA titer*: Hemagglutinin titer
MOI: Multiplicity of infection
PBST: PBS with 0.1% Tween 20
TCID_50_: 50% tissue culture infectious dose
ventilated spinner flask: Spinner flask with a small air pump that continuously supplied air to the headspace
VLP: Virus-like particle
