# Supplementary material for: Process development for pandemic influenza VLP vaccine production using a baculovirus expression system
Source: J Biol Eng. 2019 Oct 23;13:78. doi: 10.1186/s13036-019-0206-z (PMC6813129; doi:10.1186/s13036-019-0206-z)
Supplement: Supplementary file 1 — Additional file 1: Table S1. Comparison of influenza VLP production by different insect cells. [file 13036_2019_206_MOESM1_ESM.docx]

Supplement table 1. Comparison of influenza VLP production by different insect cells

| virus strain | Host cell | HA titer (HAU/ml) | Baculovirus | Reference |
| --- | --- | --- | --- | --- |
| H7N9 | Hi-5 | 512  ( about per 0.025 μg/ml total VLP protein ) | ~3.16× 10^6^  (plaque assay) | In this manuscript |
| H7N9 | Sf-9 | 64  (about per 0.025 μg/ml total VLP protein ) | ~1× 10^8^  (plaque assay) | In this manuscript |
| H7N9 | SF-9 | 512 (per 0.10 μg/ml total VLP protein) | N/A | Liu, 2015 ^1^ |
| H1N1 | Sf-9 | 335 (in pellet) | 5.85 × 10^9^  (by NSTEM) | Thompson, 2015 ^2^ |
| H1N1 | Mammalian  HEK 293 | 13 | 3.07 × 10^7^  (by NSTEM) | Thompson, 2015 ^2^ |
| H1N1 | Sf-9 | 16 | ~1× 10^8^  (plaque assay) | Krammer, 2010 ^3^ |
| H1N1 | Hi-5 | 16 | ~1× 10^6^  (plaque assay) | Krammer, 2010 ^3^ |
| H3N2 | Sf-9 | 16 | ~1× 10^8^  (plaque assay) | Krammer, 2010 ^3^ |
| H3N2 | Hi-5 | 16 | ~1× 10^6^  (plaque assay) | Krammer, 2010 ^3^ |

Reference

1. Liu YV, Massare MJ, Pearce MB, Sun X, Belser JA, Maines TR, et al. Recombinant virus-like particles elicit protective immunity against avian influenza A(H7N9) virus infection in ferrets. Vaccine 2015; 33:2152-8.

2. Thompson CM, Petiot E, Mullick A, Aucoin MG, Henry O, Kamen AA. Critical assessment of influenza VLP production in Sf9 and HEK293 expression systems. BMC Biotechnol 2015; 15:31.

3. Krammer F, Schinko T, Palmberger D, Tauer C, Messner P, Grabherr R. Trichoplusia ni cells (High Five) are highly efficient for the production of influenza A virus-like particles: a comparison of two insect cell lines as production platforms for influenza vaccines. Mol Biotechnol 2010; 45:226-34.
