# Supplementary material for: Process development for pandemic influenza VLP vaccine production using a baculovirus expression system
Source: J Biol Eng. 2019 Oct 23;13:78. doi: 10.1186/s13036-019-0206-z (PMC6813129; doi:10.1186/s13036-019-0206-z)
Supplement: Supplementary file 2 — Additional file 2: Table S2. Comparison of influenza VLPs production with shake flask and Bioreactor. [file 13036_2019_206_MOESM2_ESM.docx]

|  | Shake flask 2000 mL | Bioreactor 7.5L |
| --- | --- | --- |
| Working volume (ml) | 300 | 5000 |
| HA titer of harvest (HAU/50μl) | 512 | 512 |
| NA activities^1^ (nmol/hr/ml) | 135.2 | 141.6 |
| Western blotting^2^  (HA antiserum) | **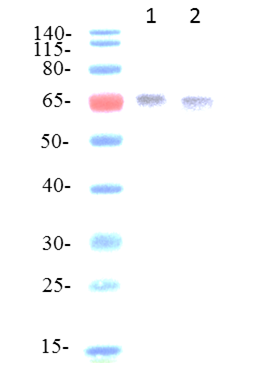** | |
| TEM^3^ | 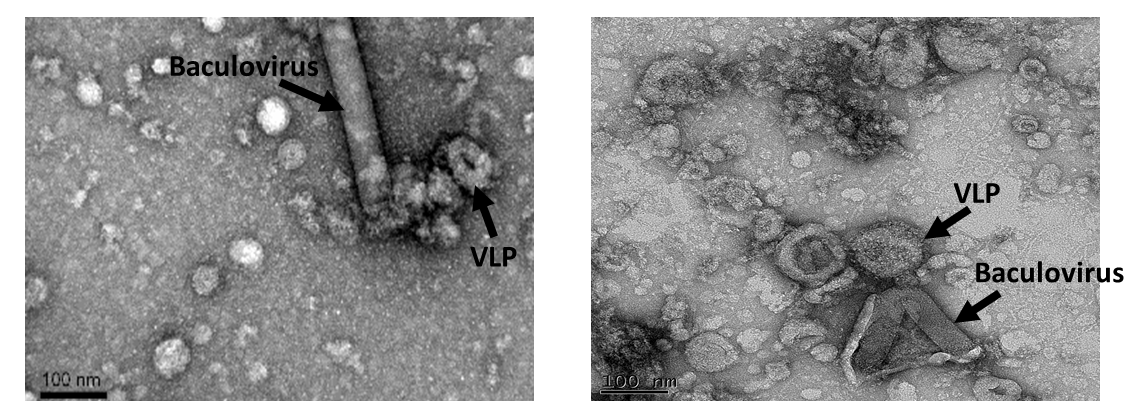 | |

Supplement table 2. Comparison of influenza VLPs production with shake flask and Bioreactor.

1. These H7N9-TW VLPs samples were purified and follow matrix and method measured NA activity.
2. These H7N9-TW VLPs samples were purified and analyzed by 10% Tris-gel SDS-PAGE. The antibody is H7N9 RG-268 antiserum from NIBSC in the western blot. The H7N9-TW VLPs was produced using the 300 ml spinner shake (left) and the 5000 ml bioreactor (right).
3. This H7N9-TW VLPs samples were harvest, then the 300 ml spinner shake (left) and the 5000 ml bioreactor (right) was observed in transmission electronic microscopic (TEM) images.
